# Activity-Dependent Increases in Quantal Size at the *Drosophila* NMJ

**DOI:** 10.3390/jdb13040038

**Published:** 2025-10-28

**Authors:** Andrew S. Powers, Petar Gajic, Ethan Rittereiser, Kavindra Dasrat, Gregory A. Lnenicka

**Affiliations:** Department of Biological Sciences, University at Albany SUNY, Albany, NY 12222, USA; andrew.stephen.powers@gmail.com (A.S.P.); petarg96@gmail.com (P.G.); eritty8@gmail.com (E.R.); kavindradasrat98@gmail.com (K.D.)

**Keywords:** *Drosophila*, NMJ, plasticity, potentiation, activity, postsynaptic

## Abstract

We examined whether an increase in synaptic activity resulted in an increase in quantal size at the neuromuscular junction (NMJ) of third-instar *Drosophila* larvae. Spontaneous miniature excitatory postsynaptic currents (mEPSCs) or miniature excitatory postsynaptic potentials (mEPSPs) were recorded before and after nerve stimulation. We found that prolonged (60 s) or brief (1.25 s) nerve stimulation produced an increase in quantal size; this appears to be a general property of these synapses since it was seen at all four muscle fibers (MFs) used in this study. The effect was examined along Is and Ib terminals by expressing GCaMP in the MF membrane and examining postsynaptic Ca^2+^ signals produced by spontaneous transmitter release. The activity-dependent increase in quantal size occurred at both Is and Ib terminals, and the increase in frequency and amplitude of quantal events at individual synaptic boutons was correlated. Both the increase in quantal size and frequency were found to be dependent upon an increase in postsynaptic Ca^2+^, based on studies in which MFs were preinjected with the Ca^2+^ chelator BAPTA (1,2-Bis(2-aminophenoxy)ethane-N,N,N′,N′-tetraacetic acid). To examine the effect of postsynaptic activity on glutamate sensitivity, we iontophoresed glutamate pulses at the NMJ and recorded the glutamate-evoked excitatory postsynaptic potentials (gEPSPs). Trains of glutamate pulses produced an increase in gEPSP amplitude; this potentiation was not seen when Ca^2+^ was eliminated from the bath or after inhibiting calmodulin or CaMKII. The activity-dependent increase in quantal size may result from an increase in postsynaptic sensitivity due to activation of CaMKII.

## 1. Introduction

Synapses play a key role in signal transmission in the brain, and the regulation of synaptic strength is important in shaping neural circuits underlying behavior. Synaptic plasticity has long been an area of interest, as synapses are capable of rapid and often long-lasting changes in efficacy. The *Drosophila* neuromuscular junction has played an important role in studies of synaptic development and plasticity as well as the molecular basis of synaptic transmission [1,2]. These studies have focused on the larval neuromuscular junction due to its accessible and identifiable synapses. The themes in synaptic plasticity that have been explored at the *Drosophila* NMJ include synaptic homeostasis and activity-dependent synaptic strengthening. Synaptic homeostatic plasticity acts to preserve synaptic strength during a variety of potential disruptions over the lifetime of the animal [3]. Activity-dependent synaptic strengthening plays a major role in the development and maintenance of synapses, as well as brain plasticity in the adult [4]. Both synaptic homeostatic plasticity and activity-dependent plasticity have short and long-term versions [3,4].

Activity-dependent synapse strengthening was first demonstrated at the vertebrate NMJ. Increased activity at the NMJ produced an increase in transmitter release, resulting in synaptic facilitation, augmentation, and post-tetanic potentiation [5]. This synaptic strengthening resulted from an increase in transmitter release and has been shown to be a general feature of synapses. Studies at the crustacean NMJ also demonstrated activity-dependent changes in transmitter release, resulting in short-term and long-term synaptic facilitation as well as greater resistance to synaptic fatigue [6]. Studies of activity-dependent synaptic strengthening in the mammalian CNS have focused on long-term potentiation (LTP) and revealed a separate mechanism for synaptic plasticity, i.e., an increase in the sensitivity of the postsynaptic membrane to the neurotransmitter. LTP produced by high-frequency, brief (seconds) synaptic activity involves a greater postsynaptic response to a single vesicle of transmitter (quantal size) triggered by an increase in postsynaptic Ca^2+^ [7,8]. In addition, it was found that increases in postsynaptic Ca^2+^ could produce multiple effects at a synapse depending on the magnitude and duration (seconds vs. minutes) of the increase [9,10].

An increase in quantal size resulting from brief stimulation has not been reported at the NMJ or shown to be a common feature of synapses. There have been occasional reports of activity-dependent increases in quantal size at the NMJ; however, these involved prolonged changes in activity [11,12]. We examined the effect of increased impulse activity on quantal size at the *Drosophila* larval NMJ and the role of postsynaptic Ca^2+^. The *Drosophila* NMJ presents a good system to examine quantal size: spontaneous miniature EPSPs (mEPSPs) and EPSCs (mEPSCs) can be directly measured since the MFs are isopotential; and these quantal events can also be visualized along the terminal with Ca^2+^ indicators in the MF [13,14,15]. Postsynaptic Ca^2+^ has previously been shown to play a role in synaptic plasticity and development at the larval NMJ. Increases in postsynaptic Ca^2+^ activate an SK channel to reduce synaptic excitation, influence the frequency of spontaneous miniature events, and enhance the growth of the NMJ during development [16,17,18,19]. A major source of postsynaptic Ca^2+^ during synaptic activity results from Ca^2+^ entering through postsynaptic glutamate receptors (GluRs). The GluRs at the *Drosophila* larval NMJ are non-NMDA receptors composed of four subunits, which include GluRIIA or GluRIIB, but not both [20]. The GluRs were found to admit Ca^2+^, and based upon their molecular structure, Ca^2+^ influx likely occurs throughGluRIIA [21]. During evoked transmitter release, single action potentials produce increases in postsynaptic Ca^2+^ that are localized to sites of transmitter release [13,14,15]. During trains of action potentials, these Ca^2+^ transients summate to further elevate the postsynaptic Ca^2+^ concentration; the increase in Ca^2+^ concentration is limited largely through Ca^2+^ extrusion by the plasma membrane Ca^2+^ pump [13].

We examined the effect of brief stimulation on quantal size at the *Drosophila* NMJ using electrophysiology and optophysiology. We found that brief high-frequency stimulation produces an increase in quantal size due to an increase in postsynaptic Ca^2+^. This was seen at multiple motor terminals and appears to be a common feature at these synapses.

## 2. Materials and Methods

Experiments were performed on MFs 4, 5, 6, and 7 in segments 3 and 4 of wandering third-instar Canton-S (CS) *Drosophila* female larvae. MFs 4, 6, and 7 are innervated by a common axon, which supplies Is (small boutons) terminals; three separate axons provide Ib (big boutons) terminals to MF4, MF5, and MF6/7 [22]. For the Ca^2+^-imaging studies, UAS-myrGCaMP6s females (gift from Dr. Troy Littleton, MIT) were crossed with male Mef2-GAL4 flies to incorporate GCaMP6 in the muscle fiber membrane [23]. All flies were reared at 25 °C on Jazz Medium (Fisher Scientific, Hampton, NH, USA). To access the NMJs, the larvae were pinned out in a physiology chamber, and after an incision through the dorsal body wall, the internal organs were removed to expose the body-wall muscles. The segmental nerves were subsequently cut, and the brain was removed. All experiments were performed in segments 3 and 4.

*Electrophysiology.* We recorded spontaneous mEPSPs or mEPSCs using sharp microelectrodes (20–30 MΩ filled with 3M KCl) connected to Axoclamp 2A or GeneClamp 500 amplifiers (Molecular Devices, Sunnyvale, CA, USA). Data were acquired (sampling rate 10 KHz) using a Digidata 1440 A digitizer (Molecular Devices) and pCLAMP 11.2 software (Molecular Devices). For voltage clamping, the holding potential was set at −60 mV for MF6 and 7, and −80 mV for MF5. Input resistance (R_in_) was measured in current clamp with a single electrode by applying square current pulses producing membrane hyperpolarizations less than 20 mV. The segmental nerve was stimulated using a suction electrode and an S11 stimulator (Grass-Telefactor, West Warwick, RI, USA). For MF4, 6, and 7, both axons were stimulated, and we recorded the compound EPSC or EPSP. For glutamate iontophoresis, electrodes were filled with 0.5 M sodium glutamate, and 15 ms current pulses were used to eject the glutamate during rapid perfusion of the preparation. In some experiments, 10 µm trifluorperazine (TFP, Sigma-Aldrich, St. Louis, MO, USA) or 5 µm KN-93 (MilliporeSigma, Burlington, MA, USA) dissolved in DMSO was added to the bath prior to the experiment. All experiments were performed in HL3 saline [24] or HL3.1 saline [25] as specified; HL3.1 has a lower Mg^2+^ concentration (4 mm) than HL3 (20 mm), resulting in greater Ca^2+^ influx [26]. All salines contained physiological Ca^2+^ levels (1.5 mm) unless otherwise indicated. All experiments were performed at room temperature (20 °C).

*BAPTA Injection.* To buffer intracellular Ca^2+^ in MF7, a sharp electrode containing 0.5 M 1,2-Bis(2-aminophenoxy)ethane-N,N,N′,N′-tetraacetic acid (BAPTA) tetrapotassium salt (Invitrogen) was inserted into MF7, and 800 ms, −30 nA hyperpolarizing pulses were delivered at 1 Hz for 5 or 10 min. Previously, it was estimated that a 5 min injection should result in a BAPTA concentration > 3 mm [18]. Once the injection protocol was complete, the BAPTA was allowed to equilibrate in the fiber for 10 min before examining the effect of nerve stimulation on mEPSC or mEPSP amplitude.

*Ca^2+^ Imaging.* The NMJs of GCaMP-expressing larvae were imaged through an Olympus BH2 upright, fixed-stage microscope equipped with epifluorescence, using a water-immersion Zeiss 40× lens (NA 0.75). Image streams were captured with a CMOS camera (PCO.edge, PCO-TECH Inc., Romulus, MI, USA) at a frame rate of 20 Hz. Excitation of GCaMP was produced by a 75 W xenon arc lamp filtered through a Lambda-10 Optical Filter Changer (Sutter Instruments, Novato, CA, USA). We used a 480 nm bandpass excitation filter, a 500 nm dichroic mirror, and a high-pass 515 nm emission filter (Chroma Technology, Brattleboro, VT, USA). Metafluor 6.1 software (Universal Imaging, Downingtown, PA, USA) was used for image acquisition as well as analysis. The average fluorescence value for each ROI was subtracted from the background (taken from an MF region without terminals) and collected for each frame.

*Data Analysis.* Spontaneous events from both the electrophysiology and Ca^2+^-imaging streams were identified and measured using MiniAnalysis (Synaptosoft). The correct identification of spontaneous events was confirmed through a manual inspection. For mEPSPs and Ca^2+^ signals, MiniAnalysis was used to measure the amplitude of individual events. For mEPSCs, MiniAnalysis was used to average spontaneous events, and the averaged mEPSCs were analyzed in SigmaPlot 12.3 (SPSS, Plover, WI, USA) to determine their amplitude, decay time constant (τ_decay_), and charge transfer. Due to higher noise levels, we found that the averaged mEPSCs gave more accurate values than the automated measurements of single events [27]. All statistical comparisons were performed using SigmaPlot 12.3.

## 3. Results

### 3.1. Nerve Stimulation Produces Potentiation of mEPSCs

**Figure 1:**
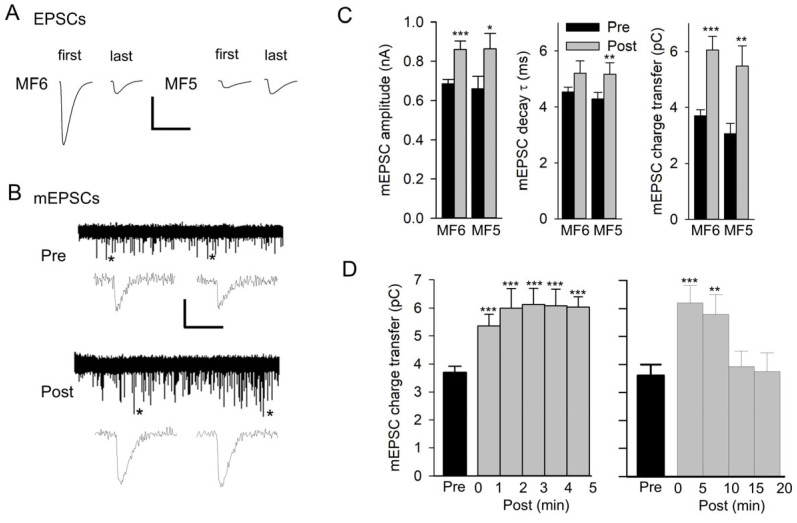
Repetitive nerve stimulation results in an increase in mEPSC size. (**A**) Representative EPSCs at the beginning (first) and end (last) of 20 Hz nerve stimulation for 1 min recorded in HL3 saline. The compound EPSC recorded from MF6 showed synaptic depression, and the EPSC recorded from MF5 showed synaptic facilitation. Calibration: 75 nA; 20 ms. (**B**) A representative experiment for MF6 showing a 5 min recording of mEPSCs before (Pre) and after stimulation (Post). mEPSCs marked with an asterisk on their right are expanded below the 5 min traces. Calibration: 1 nA; 60 s (5 min trace); 20 ms (expanded mEPSCs). (**C**) mEPSCs were averaged for each experiment, and all experiments were combined for MF6 (*n* = 10 MFs, 5 larvae) and MF5 (*n* = 6 MFs, 5 larvae). Mean values of mEPSC amplitude, decay τ, and charge transfer are shown pre and post stimulation for MF5 and MF6. Values before and after stimulation were compared using a paired *t*-test: * *p* < 0.05; ** *p* < 0.01; *** *p* < 0.001. (**D**) The time course of the increase in mEPSC size was examined for MF6 in experiments where the mEPSC charge transfer was followed for 5 min (left, *n* = 10 MFs, 5 larvae) or 20 min (right, *n* = 5 MFs, 3 larvae) after stimulation. Post values were compared to pre values using a one-way repeated measures ANOVA with a post hoc Holm–Sidak test: ** *p*< 0.01; *** *p* < 0.001.

To examine the effect of synaptic activity on quantal size, mEPSCs were recorded from the MF before and after 20 Hz nerve stimulation for 1 min in HL3 saline. For MF6, the two axons supplying the Is and Ib terminals were electrically stimulated, and the compound EPSC was recorded. The EPSC amplitude showed depression during stimulation (Figure 1A) due to the Is synapses, which show prominent synaptic depression, whereas the Ib terminal shows facilitation [28]. We also studied MF5, which only receives a single Ib terminal. This Ib terminal produced an EPSC that showed synaptic facilitation during 20 Hz stimulation (Figure 1A).

Spontaneous mEPSCs were recorded for 5 min before and after nerve stimulation and typically showed an increase in mEPSC size (Figure 1B). This increase was not due to compound mEPSCs since we did not see inflections on the rising phase of mEPSCs, indicating the summation of multiple mEPSCs. For both MF5 and MF6, the mEPSCs before and after stimulation were averaged to give a single trace, and these traces were measured to give values for amplitude, decay time constant (τ), and charge transfer (Figure 1C). As a result of stimulation, there was a significant increase in mEPSC amplitude for MF5 and MF6; the increase in the mEPSC decay τ was significant for MF5 but not for MF6. There was a significant 64% and 78% increase in charge transfer for MF6 and MF5, respectively.

To examine the time course of the increase in mEPSC size, the MF6 mEPSC charge transfer was plotted every minute post-stimulation for 5 min (Figure 1D). The mEPSC charge transfer increased during the first minute post-stimulation, and this increase persisted for 5 min. In a few cases, the mEPSCs were followed for 20 min post-stimulation, showing that the increase persisted for approximately 10 min before returning to its initial value (Figure 1D).

### 3.2. Brief High-Frequency Stimulation Produces Potentiation of mEPSPs

**Figure 2:**
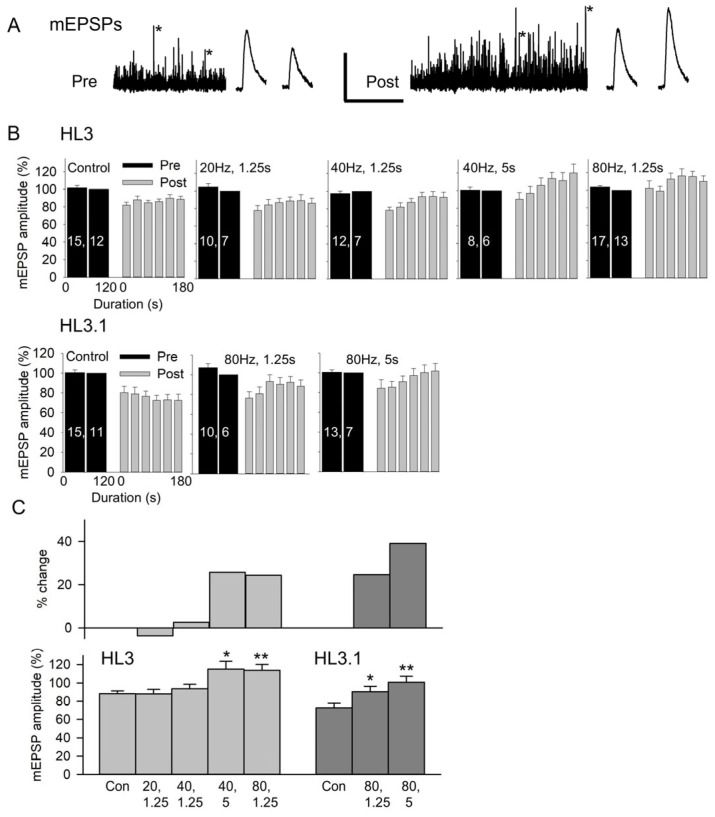
The effect of nerve stimulation on mEPSP amplitude. (**A**) Recordings of MF6 mEPSPs for 2 min before (Pre) and for 3 min after (Post) stimulation at 80 Hz for 1.25 s in HL3. mEPSPs marked with an asterisk were expanded and are shown to the right of the 2 and 3 min traces. Calibration: 2 mV; 60 s or 200 ms (expanded traces). (**B**) Combined data showing the pre- and post-mEPSP amplitudes recorded in HL3 and HL3.1 for control (Control) and stimulation (stimulation frequency, duration) experiments. All mEPSP amplitudes were normalized to the mEPSP amplitudes recorded during 60–120 s pre-stimulation. *n* values are shown on the pre bars (MFs, larvae). (**C**) To determine whether there was a stimulation-induced increase in mEPSP amplitude, we compared the post-stimulation mEPSP amplitudes to control experiments. *Bottom:* The normalized mEPSP amplitudes averaged during 90–180 s post-stimulation were compared to controls using a one-way ANOVA with a post hoc Holm–Sidak test: * *p* < 0.05, ** *p* < 0.01. *Top*: Shows the percent change in mEPSP amplitude resulting from each stimulation protocol.

We determined whether the potentiation of quantal size could be produced by briefer periods of stimulation involving higher stimulation frequencies. In these experiments, we examined a number of different stimulation paradigms and recorded mEPSPs rather than mEPSCs from MF6 since mEPSPs are easier to record. Nerve stimulation at high frequencies produced strong muscle contraction, requiring the removal of the recording electrode from the muscle during stimulation. We recorded mEPSPs for 2 min, removed the electrode from the MF, stimulated the nerve, and then reinserted the electrode and recorded mEPSPs for 3 min (Figure 2A). Experiments were performed in HL3 and HL3.1, which has a lower Mg^2+^ concentration than HL3 and allow for greater Ca^2+^ influx [26]. Control experiments were performed using the same protocol; however, the nerve was not stimulated. The effect of nerve stimulation on mEPSP amplitude was compared to the control experiment since removing and reinserting the electrode resulted in a reduction in mEPSP amplitude, especially in HL3.1.

The data for the various stimulation protocols were compared by grouping the post-stimulation mEPSP amplitudes in 30 s bins and normalizing post-stimulation to the pre-stimulation values (Figure 2B). Note that reinserting the electrode required about 5 s, so measurements were not performed for a full 30 s for the first post-stimulation bin. In HL3, the post-stimulation mEPSP amplitude increased as we increased stimulation frequency or duration. For HL3.1, the nerve was only stimulated at 80 Hz, and the post-stimulation mEPSP amplitude increased as the duration was increased from 1.25 s to 5 s. The increase in mEPSP amplitude was quantified by comparing the post-stimulation mEPSPs to the controls (Figure 2C). Here, we compared the normalized values for 90–180 s post-stimulation since the mEPSP amplitudes reach their peak during this period. In HL3, there was no significant effect of nerve stimulation at 20 Hz for 1.25 s (25 impulses) or 40 Hz for 1.25 s (50 impulses); however, mEPSP amplitude was significantly increased by 26% after stimulation at 40 Hz for 5 s (200 impulses) and by 24% after stimulation at 80 Hz for 1.25 s (100 impulses). In HL3.1, 80 Hz stimulation for 1.25 s and 5 s (400 impulses) produced a significant increase in mEPSP amplitude of 25% and 39%, respectively. Thus, the mEPSP potentiation can be produced by brief, high-frequency stimulation, and either increasing the frequency or duration of stimulation can increase the amount of potentiation.

**Figure 3:**
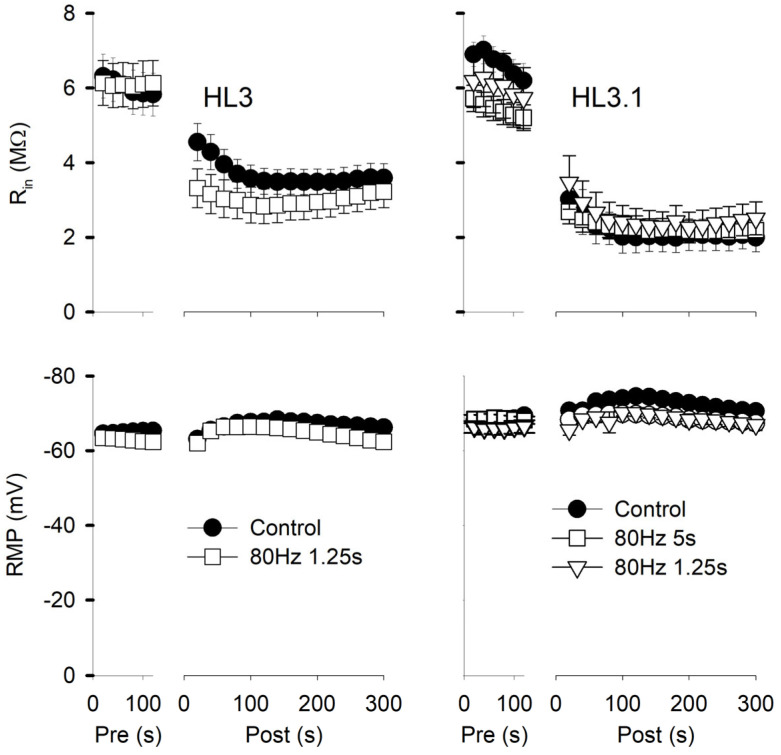
Muscle fiber input resistance (R_in_) and resting membrane potential (RMP) during two successive electrode penetrations. R_in_ and RMP were measured every 20 s during the initial penetration (Pre) and the repenetration (Post). For each treatment, the last pre value was compared to all post values using one-way repeated measures with a post hoc Holm–Sidak test. Left: Experiments were performed in HL3 with 80 Hz,1.25 s stimulation or no stimulation (Control). For R_in_, all post values were significantly less (*p* < 0.001) than pre for both control (*n* = 20 MFs, 16 larvae) and 80 Hz, 1.25 s stimulation (*n* = 12 MFs, 7 larvae). For RMP, post 80 s to 200 s values were significantly different from pre values for controls, and post 40 s to 200 s values were significantly different (*p* < 0.05) from pre values for 80 Hz, 1.25 s. Right: Experiments were performed in HL3.1. All post values for R_in_ were significantly less (*p* < 0.001) than pre values for all treatments. For RMP, none of the post values were significantly different from Pre for 80 Hz, 1.25 s (*n* = 11 MFs, 6 larvae) and 80 Hz, 5 s (*n* = 28 MFs, 17 larvae), but post 40 s to 160 s values were significantly different from pre values (*p* < 0.05) for controls (*n* = 13 MFs, 11 larvae).

It was noted that in the control experiments, there was a reduction in mEPSP amplitude after the second electrode penetration, and this was more pronounced in HL3.1 than in HL3. This reduction in mEPSP amplitude could be due to a reduction in muscle fiber input resistance (R_in_) produced by the second electrode penetration. To examine this, we measured the R_in_ and resting membrane potential (RMP) during the initial and second penetration (Figure 3). In HL3 control experiments, the R_in_ showed an initial decrease of 17% after the second penetration and continued to decline, with a final decrease of 34%. In HL3 with 80 Hz, 1.25 s stimulation, the initial decrease in R_in_ was 41% and the final decrease was 40%. In both cases, there was a significant increase in the RMP (more negative). The measurements in HL3.1 showed an even greater decrease in R_in_ after the second penetration. For the control (80 Hz, 1.25 s and 80 Hz, 5 s), the initial decline ranged from 40% to 50% and the final decrease was 50% to 70%. Only the control showed a significant increase in RMP. Thus, it appears that a reduction in R_in_ resulting from a second penetration produced the reduction in mEPSP amplitude seen in the controls. This reduction in R_in_ does not appear to result from an increased leakage conductance due to electrode damage but is likely due to an increase in K^+^ conduction (see Section 4.1).

### 3.3. mEPSP Amplitude Appears Potentiated by In Vivo Activity

**Figure 4:**
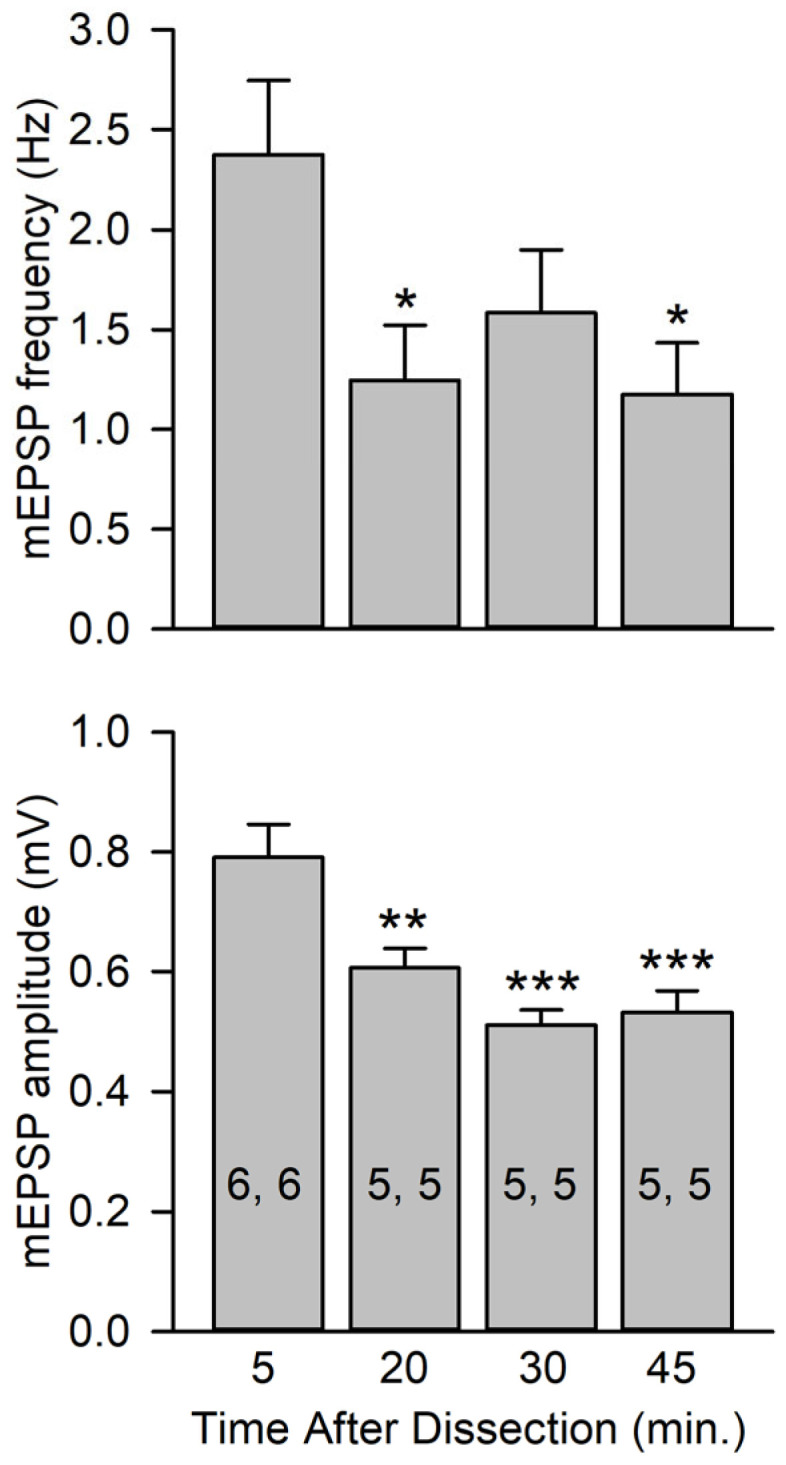
mEPSP amplitudes at various times after dissection. mEPSPs were recorded from preparations rested for various intervals after dissection; intervals were measured from the time that nerves were cut to the beginning of mEPSP recordings. The time intervals were 5, 20, 30, and 45 min (±10 s for all experiments). All experiments were performed in HL3 saline. Top: mEPSP frequencies at 20 and 45 min were significantly less than those measured at 5 min. Bottom: mEPSP amplitudes measured at 20, 30, and 45 min were significantly less than those measured at 5 min. *n* values are posted on bars (MFs, larvae) and data were analyzed using a one-way ANOVA with a post hoc Holm–Sidak test; * *p* < 0.05, ** *p* < 0.01, *** *p* < 0.001.

We examined whether the mEPSP amplitude might be normally potentiated during in vivo nerve activity by comparing mEPSP amplitudes immediately after dissection to those after a period of rest. We measured the time from cutting the nerve and eliminating impulse activity to the beginning of mEPSP recordings. We found that animals examined soon after dissection (5 min) showed a larger mEPSP amplitude than preparations rested for 20, 30, or 45 min: the mEPSP amplitude showed a significant 24% reduction when rested for 20 min and about a 35% decrease at 30 and 45 min (Figure 4). The mEPSP frequency also showed a significant decrease as the preparation rested (Figure 4). It is likely that both mEPSP amplitude and frequency were increased by prior in vivo activity.

### 3.4. Potentiation of Quantal Ca^2+^ Signals

We examined the potentiation of quantal currents using optical techniques, which allowed the potentiation to be compared between the Is and Ib synaptic terminals and along individual terminals. We examined the effect of stimulation on the amplitude of spontaneous postsynaptic Ca^2+^ signals, which arise from the entry of Ca^2+^ through postsynaptic GluRs [13,15]. Postsynaptic Ca^2+^ was detected by expressing GCaMP in the postsynaptic membrane; GCaMP is concentrated at the postsynaptic region due to the subsynaptic reticulum (SSR), an infolding of the postsynaptic membrane. All experiments were performed on MF4 since the terminals can be most easily imaged on this fiber [29]. We examined 12 Is and 12 Ib terminals with an average terminal length of 50.0 ± 6.1 µm for Is and 62.5 ± 8.0 µm for Ib.

**Figure 5:**
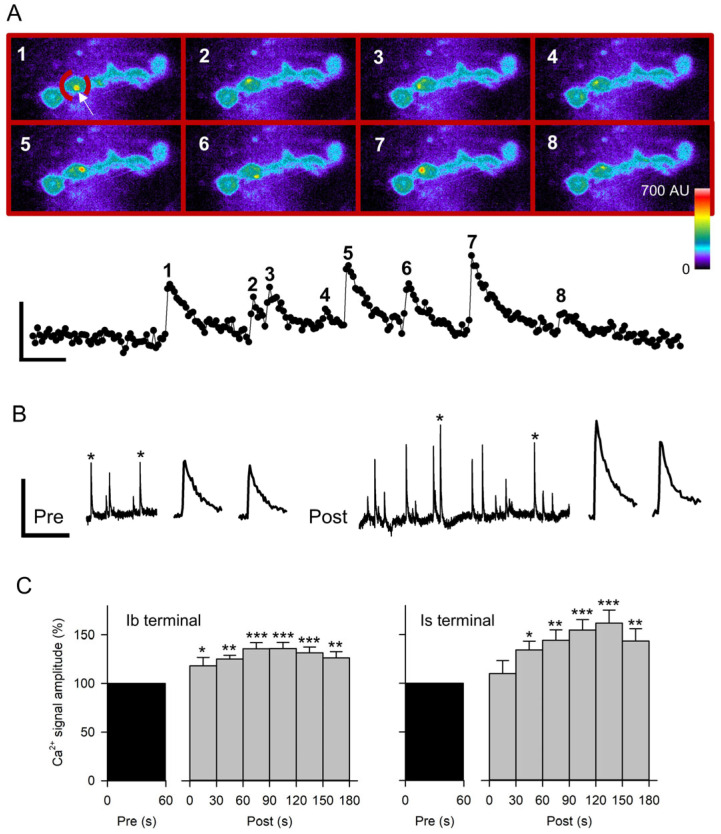
Quantal Ca^2+^ signals recorded before and after nerve stimulation. (**A**) Spontaneous Ca^2+^ signals were quantified from frames acquired at 20 Hz. Top: Pseudo-colored images of GCaMP expression in MF4 opposite an Ib terminal. Each frame shows the peak fluorescence of a spontaneous Ca^2+^ signal (white arrow in frame 1) in the ROI (broken red line in frame 1) containing a single bouton. Calibration: 15 µm. Bottom: Measurements of the average fluorescence in the ROI from a series of 282 frames. The peak amplitude of the Ca^2+^ signals was obtained from the corresponding numbered images. Calibration: 20 AU, 1 s. (**B**) Representative traces from a single bouton (ROI) showing spontaneous Ca^2+^ signals for 60 s before and 180 s after 80 Hz stimulation of the nerve for 5 s. Expanded traces of the Ca^2+^ signals marked with an asterisk are shown to the right. Calibration: 10 AU, 40 s or 2 s (expanded traces). (**C**) Combined data for Ca^2+^ signal amplitudes from experiments imaging the Ib terminals (*n* = 12 MFs, 12 larvae) and Is terminals (*n* = 12 MFs, 12 larvae) before and after nerve stimulation at 80 Hz for 5 s. The data was normalized to the pre-stimulation values. Post-stimulation values were compared to pre values before normalization using a one-way ANOVA with a post hoc Holm–Sidak test: * *p* < 0.05, ** *p* < 0.01, *** *p* < 0.001.

We measured the spontaneous Ca^2+^ signals in regions of interest (ROIs), which contained the postsynaptic GCaMP fluorescence associated with a single synaptic bouton (Figure 5A). The peak increase in the arbitrary units (AU), averaged for all pixels within the ROI, was compared pre- and post-stimulation for the same ROIs (Figure 5B). Prior to stimulation, the Ca^2+^ signal frequency per terminal length was significantly greater for Ib terminals (1.86 ± 0.36 Hz/100 µm) compared to Is terminals (0.75 ± 0.09 Hz/100 µm; *p* < 0.01, *t*-test); this is consistent with previous findings and appears to be due to fewer active zones along the Is terminals [30].

We used the paradigm 80 Hz stimulation for 5 s in HL3.1 since this produced the largest increase in mEPSP amplitude. We first repeated this stimulation in GCaMP-expressing MF4 to verify that this preparation showed the mEPSP potentiation previously seen in MF6. After stimulation of GCaMP-expressing MF4, the normalized mEPSP amplitude for 90–180 s post-stimulation (101.3 ± 7.0%; *n* = 13 MFs, 12 larvae) was significantly greater than GCaMP-expressing MF4 controls (77.6 ± 4.1%, *t*-test *p* < 0.01; *n* = 12 MFs, 7 larvae), showing a 31% increase in mEPSP amplitude. Thus, the GCaMP-expressing MF4 showed the potentiation previously seen in CS MF6. For the Ca^2+^-imaging experiments, we imaged either an Is or an Ib terminal, and both axons were stimulated. Stimulation produced a significant increase in Ca^2+^ signal amplitude for both Is and Ib terminals within the first 60 s post-stimulation that persisted for the entire 180 s (Figure 5C). The Ca^2+^ signal amplitude recorded 90–180 s post-stimulation showed a 53.4 ± 10% increase for Is terminals and a 31.5 ± 5.8% increase for Ib terminals. During this period, the Ca^2+^ signal frequency also increased 103.6 ± 19.4% for Is terminals (*p* < 0.001, paired *t*-test) and 93.1 ± 37.0% for Ib terminals (*p* < 0.01; paired *t*-test).

We compared the increase in Ca^2+^ signal amplitude and frequency at individual boutons along the terminal. We compared the most distal bouton (b1) to two more proximal boutons (b2 and b3). For the three boutons, there was no significant difference in the increase in amplitude (b1, 24.0 ± 7.7%, *n* = 21; b2, 34.1 ± 7.9%, *n* = 19; b3, 29.4 ± 7.8%, *n* = 16; *p* > 0.1, one-way ANOVA) or the increase in frequency (b1, 98.0 ± 40.1%; b2, 76.4 ± 32.3%; b3, 95.5 ± 30.8%; *p* > 0.1, one-way ANOVA). However, the percentage increase in frequency and amplitude at individual boutons was significantly correlated when comparing all boutons (r^2^ = 0.29, *n* = 72 boutons, *p* < 0.001; linear regression).

### 3.5. The Potentiation of Quantal Size Is Dependent on Increased Postsynaptic Ca^2+^ Concentration

**Figure 6:**
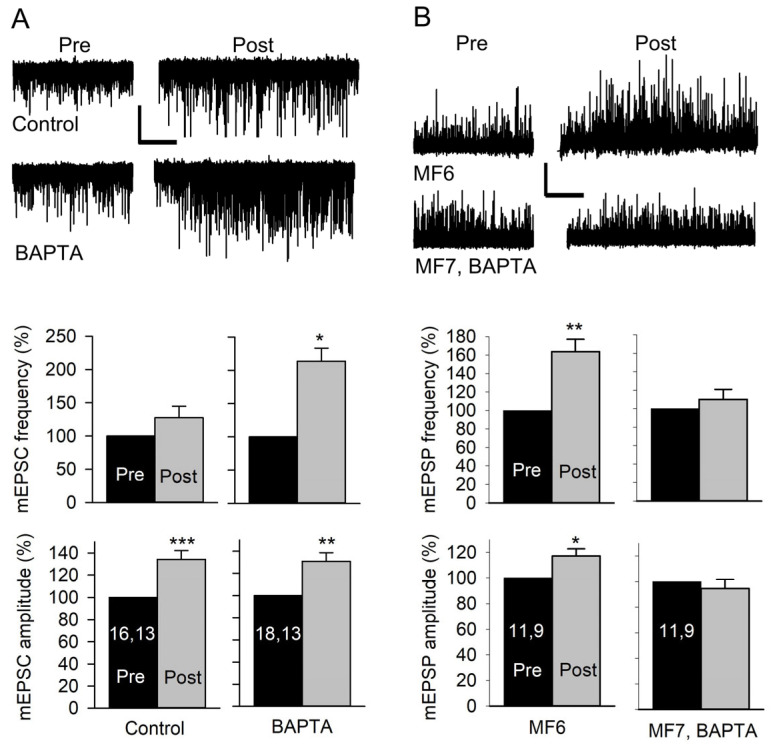
Effect of postsynaptic Ca^2+^ on the amplitude and frequency of mEPSCs and mEPSPs. (**A**) mEPSCs were recorded from MF7 for 3 min before (Pre) and for 5 min after (Post) 20 Hz nerve stimulation for 1 min in HL3. *Top:* Representative traces of mEPSCs from a Control (uninjected) MF7 and an MF7 pre-injected with BAPTA. Calibration: 1 nA; 60 s. *Bottom:* The pre- and post-EPSC amplitudes were averaged, and the combined data showed that controls did not show an increase in mEPSC frequency but showed an increase in mEPSP amplitude after stimulation. After BAPTA injection, stimulation produced an increase in both mEPSC frequency and amplitude. (**B**) mEPSPs were recorded from pairs of MF6 (uninjected) and MF7, BAPTA (preinjected with BAPTA), before and after stimulation at 80 Hz in HL3 for 1.25 s. Top: Representative mEPSP traces before (Pre) and after (Post) stimulation. Bottom: Stimulation produced an increase in mEPSP amplitude and frequency for MF6. MF7, BAPTA did not show an increase in mEPSP amplitude or frequency. Calibration: 1 mV; 60 s. *n* values are posted on bars (MFs, larvae). Pre and post amplitudes (before normalization) were compared using a paired *t*-test: * *p* < 0.05; ** *p* < 0.01; *** *p* < 0.001.

To determine whether an increase in postsynaptic Ca^2+^ concentration was required for the potentiation of quantal size, we injected the Ca^2+^ buffer BAPTA into the MF prior to performing stimulation experiments. In these experiments, we used MF7 since its small size made it easier to fill with BAPTA, and we examined mEPSC and mEPSP amplitude and frequency. First, we stimulated at 20 Hz for 1 min and recorded mEPSCs for 3 min pre-stimulation and 5 min post-stimulation in HL3. In control experiments, stimulation produced an increase in mEPSC amplitude but no change in mEPSC frequency (Figure 6A). When MF7 was preinjected with BAPTA for 5 min, there was an increase in both mEPSC amplitude and frequency. The effect of BAPTA injection on mEPSC frequency is consistent with a previous study where prolonged Ca^2+^ influx was found to depress mEPSC frequency [18]. It appeared that BAPTA injection lowered postsynaptic Ca^2+^ enough to reduce the depression of mEPSP frequency but not enough to prevent the increase in mEPSP amplitude. To allow for more effective buffering of Ca^2+^, we repeated the experiments using a briefer stimulation protocol. The nerve was stimulated at 80 Hz for 1.25 s in HL3, and mEPSPs were recorded from MF6 and MF7 pairs; MF6 and MF7 are adjacent and innervated by the same motor axons. In controls, there was an increase in mEPSP amplitude and frequency for both MFs. The mEPSP amplitude increased by 16.6 ± 7.2% in MF6 (*p* < 0.05, paired *t*-test) and 17.6 ± 4.5% (*p* < 0.01, paired *t*-test; *n* = 10 MFs, 8 larvae) in MF7. The mEPSP frequency increased by 34.0 ± 7.6% in MF6 (*p* < 0.01, paired *t*-test) and 46.8 ± 7.1% (*p* < 0.001, paired *t*-test) in MF7. We repeated this experiment after injecting MF7 with BAPTA. We found that BAPTA injection prevented the increase in mEPSP amplitude and frequency in MF7; however, the previous increase in mEPSP amplitude and frequency was still seen in uninjected MF6 (Figure 6B). Thus, BAPTA injection blocked both the increase in mEPSP amplitude and frequency produced by brief stimulation.

### 3.6. Activation of GluRs Produces a Ca^2+^-Dependent Increase in Glutamate Sensitivity

**Figure 7:**
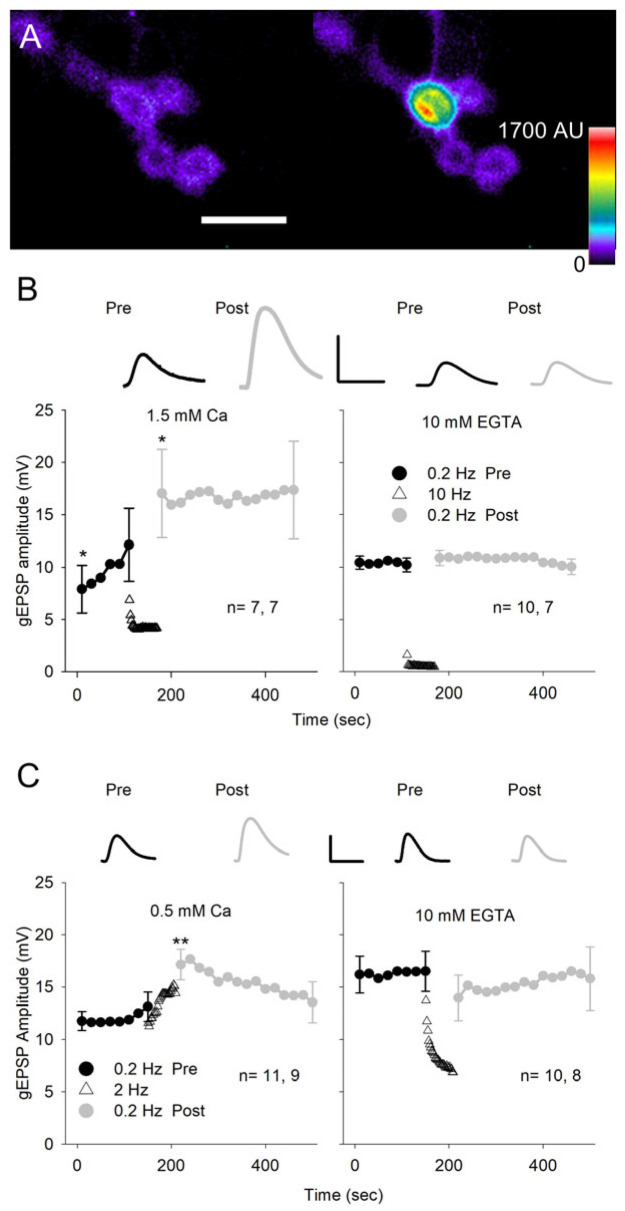
Glutamate iontophoresis. (**A**) Glutamate iontophoresis on a GCaMP-expressing MF4 NMJ showing activation of a single bouton. Pseudo-color images of postsynaptic GCaMP fluorescence before (left) and during glutamate iontophoresis (right). Calibration: 10 µm. (**B**) gEPSPs were generated at 0.2 Hz for 150 s before (Pre) and at 0.2 Hz for 300 s after (Post) conditioning pulses that were delivered at 10 Hz for 60 s. For each experiment, every 4 gEPSP amplitudes were averaged to give a single value. Experiments were performed in HL3 saline with 1.5 mm Ca^2+^ (left) or containing 0 Ca^2+^ with 10 mm EGTA (right). In 1.5 mm Ca^2+^, the gEPSP amplitude increased during both 0.2 Hz stimulation and 10 Hz stimulation. In 10 mm EGTA, there was no increase in gEPSP amplitude during stimulation. (**C**) The conditioning glutamate pulses were delivered at 2 Hz, and experiments were performed in either 0.5 mm Ca^2+^ (left) or 10 mm EGTA saline (right). In 0.5 mm Ca^2+^, the gEPSP amplitude increased as a result of 2 Hz conditioning stimulation. In 10 mm EGTA, there was no increase in gEPSP amplitude as a result of 2 Hz stimulation. Inserts: Representative gEPSPs are shown for the first response during 0.2 Hz stimulation before (Pre) and after (Post) conditioning. Calibration: B- 20 mV, 70 ms; C- 10 mV, 125 ms. *n* values are shown on each graph (MFs, larvae). For all experiments, the last pre-gEPSP was compared to the first pre-gEPSP and the first post-gEPSP using a one-way repeated measures ANOVA with post hoc Holm–Sidak test: * *p* < 0.05; ** *p* < 0.01.

An increase in quantal size could be due to greater sensitivity of the postsynaptic membrane to glutamate. In an earlier study, the increased glutamate sensitivity during LTP was demonstrated by repetitive application of glutamate to spines in the mammalian hippocampus [31]. In our study, we applied repetitive glutamate pulses to the NMJ on MF4 using iontophoresis (Figure 7A). With a glutamate electrode positioned over a synaptic bouton, we applied 15 ms glutamate pulses and recorded the glutamate-evoked EPSPs (gEPSPs). We adjusted the amplitude of the glutamate pulse to give an amplitude of 10–15 mV and examined the effect of pulses delivered at low and moderate frequencies on gEPSP amplitude. Initially, experiments were performed in HL3.0 with 1.5 mm Ca^2+,^ and test pulses were delivered at 0.2 Hz for 2 min before and 5 min after the conditioning stimulation delivered at 10 Hz for 1 min (Figure 7B). The gEPSP amplitude showed a significant increase even during the first 0.2 Hz test pulses, and the 10 Hz conditioning pulses produced a further increase seen immediately after conditioning. Subsequently, the 0.2 Hz, 5 min stimulation did not further increase gEPSP amplitude, suggesting that the potentiation had reached saturation. In a few experiments (4), the post-conditioning 0.2 Hz train was continued for 10 min, and the gEPSP amplitude remained elevated. These results clearly show that activation of the GluRs can produce an increase in postsynaptic sensitivity to glutamate. To determine whether this effect was Ca^2+^-dependent, the experiments were repeated in Ca^2+^-free saline with EGTA (Figure 7B). Under these conditions, there was no increase in gEPSP amplitude resulting from 0.2 Hz or 10 Hz stimulation.

We changed our protocol by lowering the external Ca^2+^ to 0.5 mm and reducing the conditioning stimulation from 10 to 2 Hz (Figure 7C). This eliminated the increase in gEPSP amplitude seen during the initial test pulses and reduced the decrease in gEPSP amplitude seen during the 10 Hz stimulation, which presumably resulted from desensitization of the GluRs. Again, the conditioning stimulation produced a significant increase in gEPSP amplitude, and when we repeated the experiment in Ca^2+^-free saline with EGTA, the potentiation of the gEPSP was eliminated. Thus, repetitive activation of the GluRs produces an increase in glutamate sensitivity that was dependent upon Ca^2+^ influx.

**Figure 8:**
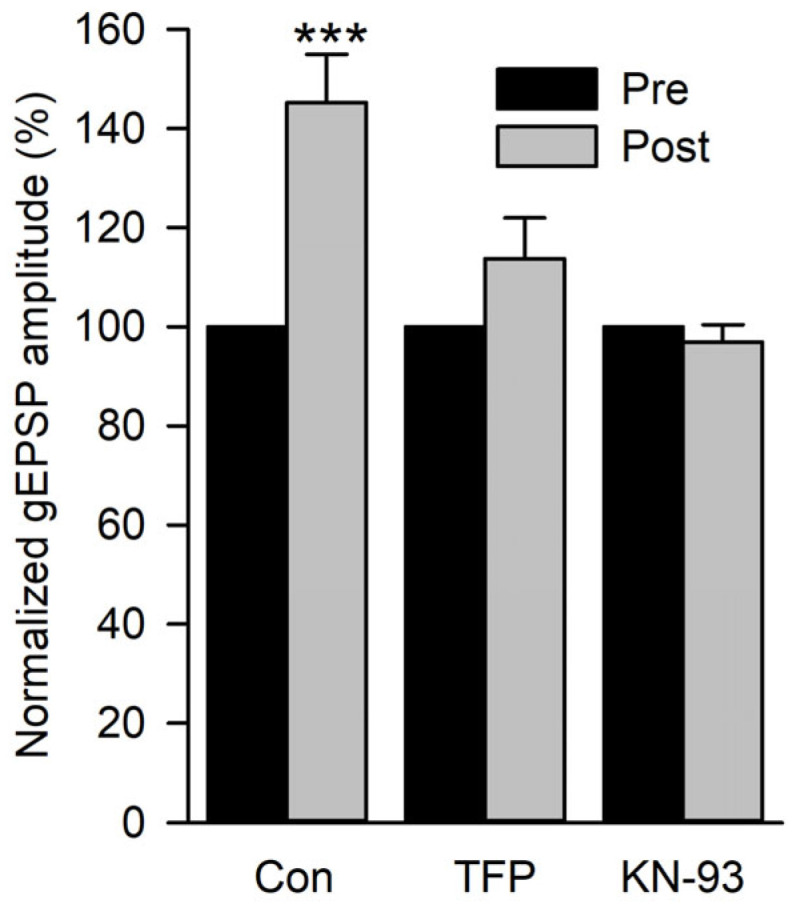
The effect of calmodulin and CamKII inhibitors on the potentiation of gEPSP amplitude. The potentiation of gEPSPs produced by glutamate conditioning pulses delivered at 2 Hz was compared for control (Con), TFP, and KN-93 experiments. All post-conditioning values were normalized to their pre-values. Control experiments (*n* = 11 MFs, 9 larvae) showed a significant increase in gEPSP amplitude, but TFP (*n* = 12 MFs, 6 larvae) and KN-93 (*n* = 19 MFs, 6 larvae) experiments did not. Pre-gEPSP amplitudes were compared to post-gEPSP amplitudes using a paired *t*-test before normalization. *** *p* < 0.001.

To test whether calmodulin and CaMKII were involved in the increase in gEPSP amplitude, we applied a calmodulin inhibitor (10 µM TFP) or CaMKII inhibitor (5 µm KN-93) before performing the 2 Hz conditioning experiment in 0.5 mm Ca^2+^ as previously described (Figure 7C). The mean gEPSP amplitude during the 150 s pre-conditioning was compared to the mean gEPSP amplitude recorded during the first 60 s post-conditioning in control, TFP, and KN-93 experiments (Figure 8). Conditioning produced a significant increase in gEPSP amplitude in controls but not in TFP or KN-93 experiments. Thus, it appears that the increase in postsynaptic Ca^2+^ produced an increase in glutamate sensitivity by activating CaMKII.

## 4. Discussion

### 4.1. An Activity-Dependent Increase in Quantal Size Was Demonstrated by Electrophysiological Measurements

We found that prolonged stimulation (20 Hz, 1 min) or brief, high-frequency stimulation (80 Hz, 1.25 s) can produce an increase in quantal size, which persists for up to 10 min The increase in quantal size was seen by measuring spontaneous mEPSCs, mEPSPs and postsynaptic Ca^2+^ signals and must result from greater synaptic current produced by a single vesicle of transmitter. In a very early study of the rat NMJ, there appeared to be an increase in mEPSP amplitude after repetitive stimulation; however, this was attributed to summation of coincident mEPSPs due to an increase in mEPSP frequency [32]. We have examined mEPSP and mEPSCs waveforms post-stimulation and did not find evidence for this. In fact, we saw this increase in mEPSC amplitude in the absence of an increase in frequency when we stimulated the nerve at 20 Hz for 1 min. This effect likely occurs at all *Drosophila* larval neuromuscular synapses since it was observed at every MF (4, 5, 6, and 7) examined in this study.

An increase in quantal size produced by brief stimulation has not previously been reported at the NMJ, although a few studies have reported an increase in quantal size after more prolonged activity. At the rat NMJ, 1 h of in vivo nerve stimulation resulted in an increase in mEPSP amplitude, apparently due to an increase in postsynaptic sensitivity to acetylcholine [12]. An increase in mEPSC amplitude was seen at the frog NMJ after 2 h of in vivo stimulation of the motor axons [11]. At the *Drosophila* larval NMJ, 40 min of fast larval crawling resulted in an increase in the amplitude of mEPSPs due to an increase in synaptic vesicle size [33]. Note that our stimulation was similar to the brief stimulation (100 Hz, 1 s) used to produce LTP, although the effects of LTP are more long-lasting [34].

It seems likely that this activity-dependent potentiation of quantal size occurs in vivo during normal motor activity. In dissected preparations, the Ib terminals fire at frequencies up to 40 Hz [35]. Based upon our studies, this frequency would be high enough to produce the potentiation of quantal size. In addition, the frequencies in vivo may be higher than 40 Hz since the waves of muscle contractions seen in dissected preparations are generally slower than observed in the intact animal [36]. Our findings show that mEPSP amplitude and frequency recorded 5 min after dissection are larger than 20 min post-dissection. mEPSP amplitude and frequency were likely increased by high larval locomotor activity immediately prior to dissection and subsequently declined in the absence of activity. We assume that during normal locomotor activity, the synapses are strengthened by this potentiation of mEPSP amplitude, facilitating their movements. During quiescent periods, a decrease in the size and frequency of spontaneous quantal events may reduce energy consumption.

Do these changes in quantal size result in corresponding changes in synaptic strength, given the mechanisms for synaptic homeostasis found at these synapses? Indeed, synaptic homeostasis appears to operate at the time scale examined in this study: the application of philanthotoxin-433 (PhTx) reduces mEPSP amplitude and triggers a compensatory increase in transmitter release within 10 min [37]. However, this acute form of synaptic homeostasis appears to result from PhTx binding to the postsynaptic receptor rather than a decrease in mEPSP amplitude [38,39], suggesting that it is unlikely to occur during these physiological changes in mEPSP amplitude. In addition, it does not appear that a postsynaptic change increasing quantal size at these synapses results in a decrease in transmitter release [21,40], indicating that an increase in synaptic strength resulting from an increase in glutamate sensitivity would not be reversed by homeostatic plasticity.

An unexpected technical finding in these studies was that electrode repenetration of MFs produced a substantial decrease in R_in_, especially in HL3.1 saline. This appears to be a direct result of an increase in membrane conductance rather than an increase in electrode leakage conductance. An increase in leakage conductance would not be ion-specific, and therefore, it would lead to a reduction in RMP. However, the RMP was never observed to decrease, and in most cases, it increased. This increase in RMP points to an increase in membrane conductance for K^+^. The MFs have a substantial Ca^2+^-dependent K^+^ conductance [17], and it may be that Ca^2+^ leaking in during repenetration activated this K^+^ conductance. Note that this effect could impact previous measurements of the electrode leak resistance produced by MF penetration [27]. Also, this finding demonstrates that a large membrane potential cannot always be used to indicate a good electrode penetration for these MFs.

### 4.2. The Activity-Dependent Increase in Quantal Size Was Examined with Optophysiology

The postsynaptic Ca^2+^ signals produced by spontaneous transmitter release were imaged using GCaMP expressed in the postsynaptic membrane. The amplitude of these spontaneous Ca^2+^ signals should be proportional to the amount of Ca^2+^ entering through the GluRs and thus represent the magnitude of the mEPSC. We found that stimulation at 80 Hz for 5 s produced an increase in Ca^2+^ signal amplitude for both Ib and Is terminals. The Is and Ib terminals show differences in basal transmitter release [30], synaptic facilitation/depression [28], and homeostatic plasticity [30,41]; however, we find that they both show an activity-dependent potentiation of quantal size. The potentiation of Ca^2+^ signal amplitude and frequency seen at individual boutons was not influenced by bouton position. More distal boutons along the Ib terminals on MF6 show greater transmitter release and presynaptic Ca^2+^ influx [14,29], which could lead to greater potentiation of quantal size and frequency. However, the MF4 terminals used for Ca^2+^ imaging in this study did not show the proximal to distal differences in transmitter release or presynaptic Ca^2+^ influx seen on MF6, possibly due to their shorter length [15,29].

There was a correlation between the increase in Ca^2+^ signal frequency and amplitude seen at individual boutons. Since both effects are dependent on an increase in postsynaptic Ca^2+^, this might result from variability in the increase in postsynaptic Ca^2+^ along the terminal. In the mammalian CNS, LTP and the associated increase in glutamate sensitivity were found to be synaptic-spine specific [31]; presumably, the spines act to compartmentalize postsynaptic Ca^2+^ and prevent the spread of LTP. At the larval NMJ, bouton/synapse specificity could result from the SSR, which would restrict the diffusion of Ca^2+^ along the postsynaptic membrane. Also, it seems likely that the increase in quantal size and frequency occurred at only a subset of active zones. It was previously shown that some active zones show spontaneous transmitter and little or no evoked release, while others show mainly evoked release [15,42]. In either case, one might not see an increase in the amplitude or frequency of spontaneous quantal events at these active zones.

### 4.3. The Potentiation of mEPSP Amplitude Is Dependent upon an Increase in Postsynaptic Ca^2+^

The role of postsynaptic Ca^2+^ in the potentiation of mEPSP and mEPSC amplitude was examined by pre-injecting BAPTA into the muscle fiber. BAPTA injection did not eliminate the potentiation produced by 60 s of stimulation, but it blocked the potentiation produced by 1.25 s of stimulation. It seems likely that the BAPTA became saturated during prolonged synaptic activity but was more effective at buffering Ca^2+^ during the brief stimulation and blocked the potentiation of quantal size. The effect of BAPTA injection on mEPSP frequency was more complex. Brief stimulation increased mEPSP frequency, and this was blocked by preinjecting the MF with BAPTA; this was consistent with an earlier finding [19]. Stimulation for 60 s did not produce a significant change in mEPSP frequency; however, when the muscle was preinjected with BAPTA, there was an increase in mEPSP frequency. These experiments were performed in HL3; a previous study performed in HL3.1, which presumably produced greater Ca^2+^ influx, found that 60 s of stimulation resulted in a decrease in mEPSC frequency [18].

Together, the results indicate that a brief increase in postsynaptic Ca^2+^ produces an increase in frequency, whereas larger and/or more prolonged Ca^2+^ increases can depress frequency. Prolonged stimulation resulting in a decrease in mEPSP frequency was previously reported at the crayfish NMJ [43]. It is unclear whether the reduction in mEPSP frequency produced by prolonged substantial Ca^2+^ influx results from less transmitter release or a postsynaptic effect. There have been reports of missing mEPSPs (spontaneous transmitter release not leading to the generation of mEPSPs) and the possible involvement of the SSR [44]. It may be that an increase in Ca^2+^ alters membrane conduction in the SSR and shunts the synaptic current. These differential effects of postsynaptic Ca^2+^ are not surprising since increases in postsynaptic Ca^2+^ can produce multiple effects at mammalian CNS synapses depending on the magnitude and duration of the increase [9,10].

We found that for motor terminals that contacted both MF6 and 7, the increase in amplitude and frequency was only blocked at terminals innervating the MF preinjected with BAPTA (MF7). For the Ca^2+^-dependent increase in frequency, postsynaptic Ca^2+^ acts via synaptotagmin 4 to release a retrograde signal to increase spontaneous transmitter release [16,19]. It appears that the retrograde signals promoting increased spontaneous transmitter release only act locally since these terminals travel along the cleft between MF6 and 7, and a single terminal branch can innervate both MFs. Our results are similar to studies showing retrograde induction of synaptic homeostasis at terminals innervating MF6 but not those from the same axon innervating MF7 [45]. The muscle-specific block of the increase in amplitude is consistent with our findings that the increase in amplitude is likely due to greater sensitivity of the muscle to glutamate (below).

### 4.4. Glutamate Iontophoresis Produced a Ca^2+^-Dependent Increase in Glutamate Sensitivity

We found that repeated application of glutamate at the NMJ resulted in an increase in the sensitivity of the membrane to subsequent glutamate pulses. This increase in glutamate sensitivity was Ca^2+^-dependent since lowering extracellular Ca^2+^ reduced the potentiation, and eliminating extracellular Ca^2+^ blocked the potentiation. It seems likely that the Ca^2+^-dependent increase in mEPSP amplitude also resulted from this increase in glutamate sensitivity; however, glutamate pulses produced their effects at much lower stimulation frequencies than seen for nerve stimulation. This difference likely resulted from greater activation of GluRs by glutamate iontophoresis compared to nerve stimulation. Typically, nerve stimulation produces a compound EPSP of about 30 mV resulting from activation of approximately 50 (Ib + Is) boutons [46,47]. During glutamate iontophoresis, activation of GluRs at a single bouton produced a gEPSP of approximately 10 mV. Therefore, the number of GluRs activated at a single bouton during ionotophoresis was about 15-fold the number activated during nerve stimulation; individual glutamate pulses would have produced large increases in postsynaptic Ca^2+^, possibly equivalent to that produced by repetitive nerve stimulation.

The increase in glutamate sensitivity could result from an increase in the number of GluRs or activation of existing ones. During LTP, elevated postsynaptic Ca^2+^ produces an increase in the unitary conductance and number of postsynaptic GluRs [48,49]. At the larval NMJ, there is also evidence for changes in receptor number resulting from changes in activity. During early development, blocking nerve activity prevented the clustering of GluR at the postsynaptic membrane [50]. GluRs were found to preferentially cluster opposite active zones, showing high transmitter release [51], and the amount of transmitter release also influenced the relative proportion of GluRIIA and GluRIIB [52]. In addition, an increase in larval locomotion or stimulation of the NMJ in vivo using channelrhodopsin-2 produced an increase in GluRIIA number at the postsynaptic membrane [53,54]. The activity-dependent increase in quantal size we observed occurred rapidly (2–3 min.) after a brief stimulation; this could argue for activation of existing GluRs, although it appears that GluR exocytosis and lateral diffusion to the synapse can also occur within this timeframe [55,56].

We found that the calmodulin inhibitor TFP or the CaMKII inhibitor K-93 blocks the increase in gEPSP amplitude during repetitive glutamate pulses. Thus, CaMKII appears to be involved in this increase in glutamate sensitivity and could be responsible for the activity-dependent increase in quantal size. Activation of CaMKII might increase the density and/or unitary conductance of GluRs, as has been reported during LTP at mammalian synapses [49,57]. At the larval NMJ, CaMKII is found at the postsynaptic density [58] and was shown to function during synaptic homeostasis [59] and synaptic growth [60]. Thus, postsynaptic CaMKII may have multiple functions at the *Drosophila* NMJ. This is not unusual since postsynaptic CaMKII plays a role in both LTP and synaptic homeostasis at mammalian synapses; here, the different functions of CaMKII appear to be related to its multiple isoforms and splice variants, as well as to the differences in cellular localization [61].

## Data Availability

The original contributions presented in this study are included in the article. Further inquiries can be directed to the corresponding author(s).
